# Correction: Stimuli-responsive Zn(ii) complexes showing the structural conversion and on/off switching of catalytic properties

**DOI:** 10.1039/d5ra90021b

**Published:** 2025-03-25

**Authors:** So Hyeon Kwon, Sunwoo Lee, Jacopo Tessarolo, Haeri Lee

**Affiliations:** a Department of Chemistry, Hannam University 34054 Republic of Korea haeri.lee@hnu.ac.kr; b Department of Chemistry, Chonnam National University Gwangju 61186 Republic of Korea jacopo@jnu.ac.kr

## Abstract

Correction for ‘Stimuli-responsive Zn(ii) complexes showing the structural conversion and on/off switching of catalytic properties’ by So Hyeon Kwon *et al.*, *RSC Adv.*, 2024, **14**, 32655–32660, https://doi.org/10.1039/D4RA06058J.

The authors regret the omission of a funding acknowledgement in the original article. This acknowledgement is given below.

This work was financially supported by the National Research Foundation of Korea (NRF) funded by the Ministry of Science and ICT (MSIT) (grants NRF-2021R1C1C1013037 and RS-2024-00348192), by the Korea Institute for Advancement of Technology (KIAT) grant funded by the Korea Government (MOTIE, RS-2024-00409639), by the Hannam University Research Fund (2022A214), by Chonnam National University (grant number: 2023-0917-01), and by the “Regional Innovation Strategy (RIS)” through the NRF funded by the Ministry of Education (MOE) (2021RIS-002). S. H. Kwon is thankful for support from the Basic Science Research Program through the National Research Foundation of Korea (NRF) funded by the Ministry of Education (RS-2024-00466289). H. L. thanks Dr Dongwook Kim from the Institute of Basic Science (IBS) and Jihun Han from Pusan National University for the SCXRD data collection.

The Royal Society of Chemistry apologises for these errors and any consequent inconvenience to authors and readers.

